# Arterial Dissection of an Anomalous Right Vertebral Artery Arising From the Right Common Carotid Artery Associated With an Acute Right Middle Cerebral Artery Stroke

**DOI:** 10.7759/cureus.25850

**Published:** 2022-06-11

**Authors:** Allen Mao, Hoang D Duong

**Affiliations:** 1 Diagnostic Radiology, HCA Florida North Florida Hospital, Gainesville, USA; 2 Interventional Neuroradiology, HCA Florida North Florida Hospital, Gainesville, USA

**Keywords:** middle cerebral artery (mca), dissection, vertebral artery, stroke, mca

## Abstract

In rare cases, the right vertebral artery can have its origin from the right proximal common carotid artery. This anatomical variant can be incidental, but if trauma to the vertebral artery occurs, there can be devastating neurological deficits. Our patient was an adult female who initially presented with new-onset left arm weakness and dysarthria. After a CT angiogram and ultrasound imaging were performed, she was found to have an acute right middle cerebral artery (MCA) stroke in association with dissection of the anomalous right vertebral artery. The patient underwent urgent mechanical thrombectomy in the right MCA and thrombolysis in cerebral infarction (TICI) grade 3 recanalization was successfully achieved. The patient made a complete recovery with no neurological sequelae. A follow-up CT angiogram after six months showed resolution of the dissection and restored patency of the right vertebral artery.

## Introduction

The anomalous origin of the right vertebral artery from the right proximal common carotid artery (CCA) is an extremely rare anatomical variant, which, in most cases, is incidental. The incidence of this anomaly is higher when there is an aberrant right subclavian artery [1]. We highlight the hospital course of a patient who presented with an acute right middle cerebral artery (MCA) stroke in association with dissection of the anomalous right vertebral artery.

Typically, the right vertebral artery originates from the right subclavian artery and ascends posterior to the right internal carotid artery through the transverse foramina of the cervical spine [2]. It has numerous branches, the largest of which is the posterior inferior cerebellar artery, which supplies a portion of the cerebellum and merges with the contralateral vertebral artery to continue as the basilar artery [2]. Injury to the vertebral arteries would normally lead to posterior circulation symptoms such as visual disturbances and gait problems. However, when the right vertebral artery arises from the right CCA instead and is injured, a massive stroke can occur, leading to contralateral hemiparesis and speech deficits [3].

## Case presentation

Our patient is a 36-year-old female with a past medical history of right-sided migraines and frequent chiropractor neck manipulations who presented with acute left arm weakness and dysarthria. She reported that her symptoms first began during a routine gym workout after performing glute-ham developer sit-ups, where her head was whipping back and forth. After this exercise, she could not stand, and her trainer noticed her left arm was lagging and her speech was slurred.

**Figure 1:**
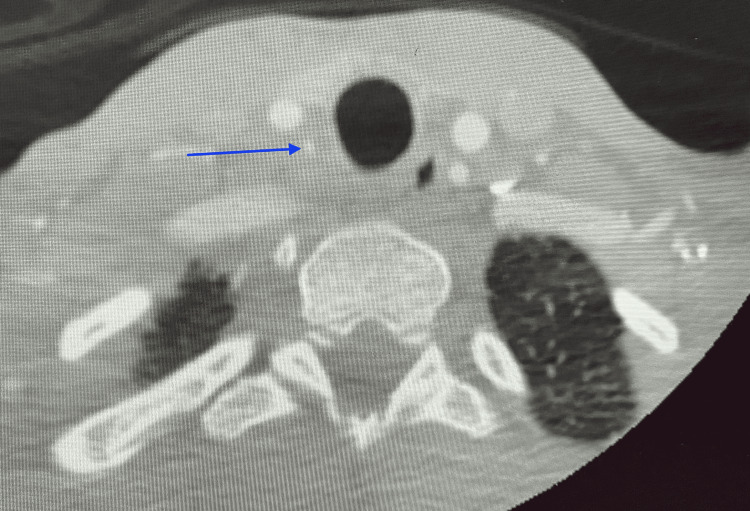
CT angiogram of the head and neck showing aberrant right vertebral artery CT angiogram of the head and neck showed an occlusion of the corresponding M2 branch and an incidental finding of an aberrant right vertebral artery arising from the right proximal common carotid artery, which appeared to be severely stenosed in its proximal cervical segment (blue arrow).

**Figure 2:**
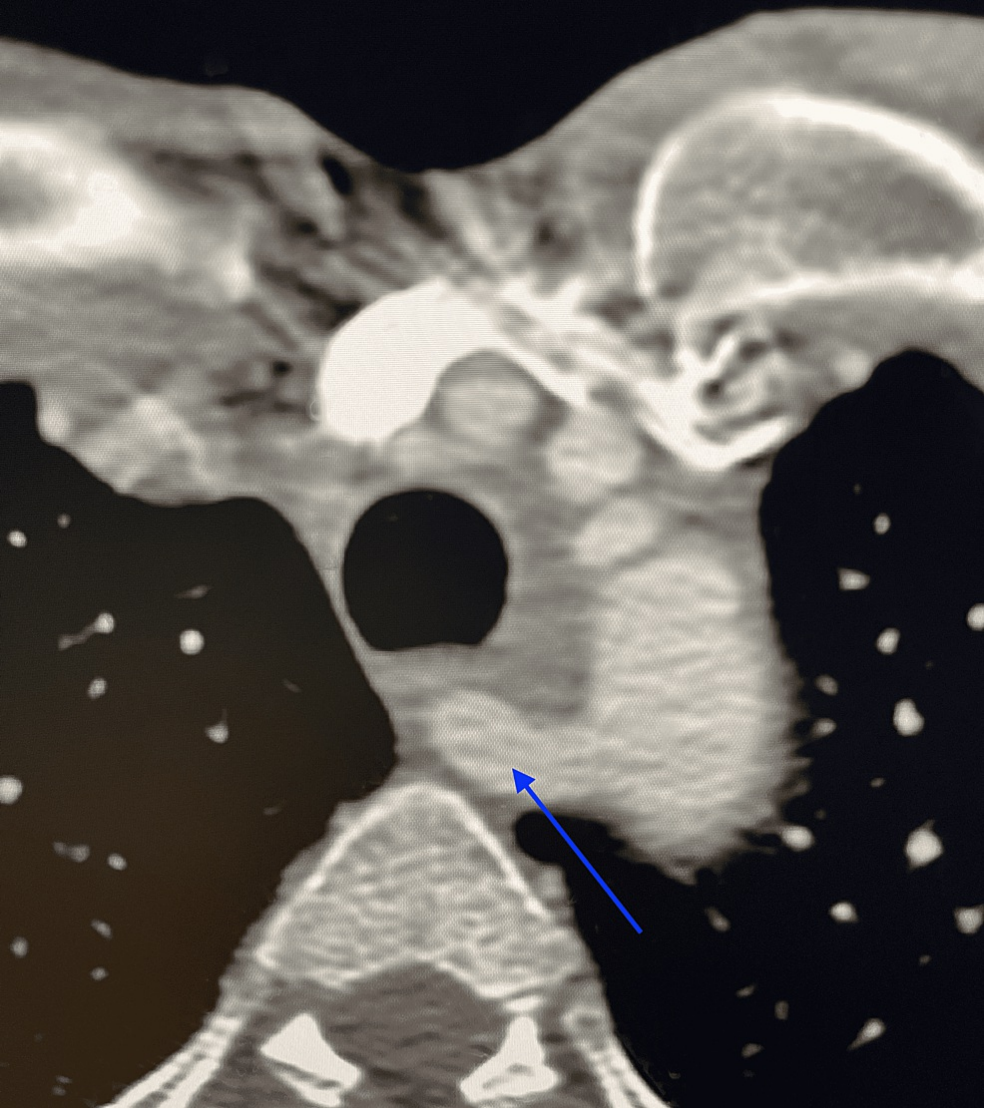
CT angiogram of the head and neck showing aberrant right subclavian artery CT angiogram of the head and neck showed an aberrant right subclavian artery (blue arrow) arising directly from the aortic arch distal to the left subclavian artery and traversing posterior to the trachea and esophagus.

**Figure 3:**
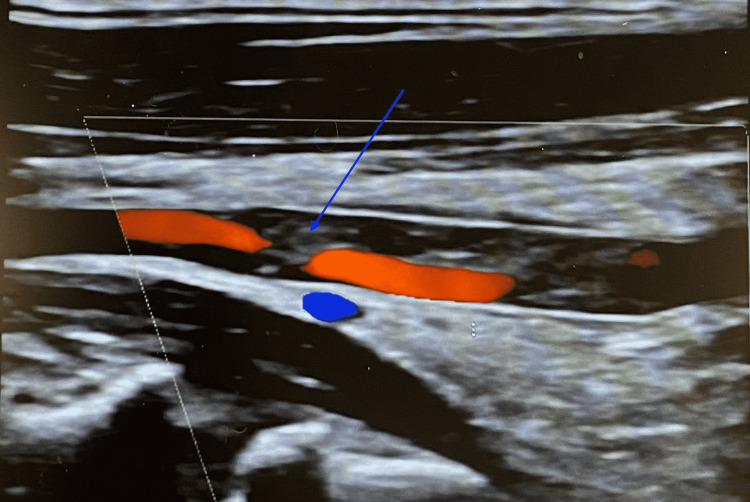
Color Doppler ultrasound of the right vertebral artery Color Doppler ultrasound showed an intraluminal thrombus (blue arrow) in the right vertebral artery, likely related to an underlying dissection.

She was immediately sent to the emergency room for further workup. The patient was given an intravenous tissue plasminogen activator (tPA) within the 4.5-hour window of symptom onset. CT perfusion imaging revealed an ischemic penumbra volume of 35 ml in the distribution of the posterior M2 division of the right MCA. CT angiography of the head and neck confirmed occlusion of the corresponding M2 branch and an incidental finding of an aberrant right vertebral artery arising from the right proximal CCA, which appeared to be severely stenosed in its proximal cervical segment (Figure 1). There was also an aberrant right subclavian artery arising directly from the aortic arch distal to the left subclavian artery and traversing posterior to the trachea and esophagus (Figure 2). Intraluminal thrombus in the right vertebral artery likely related to an underlying dissection was visualized on color Doppler sonography (Figure 3).

**Figure 4:**
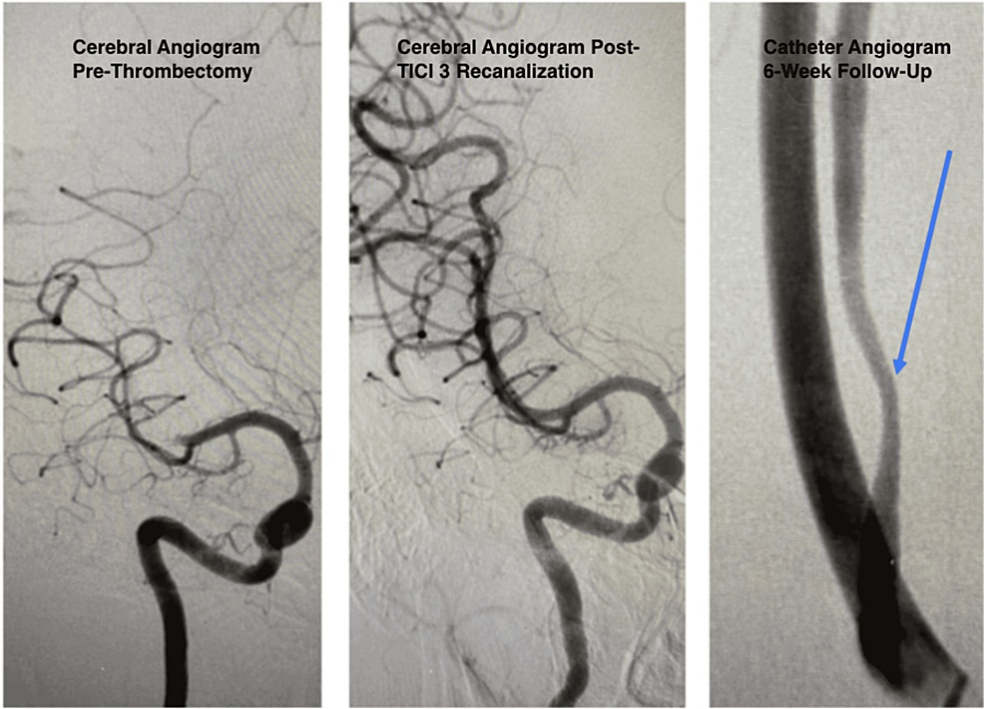
Cerebral angiogram pre-thrombectomy, post-thrombolysis in cerebral infarction (TICI) grade 3 recanalization, and at six-week follow-up The patient underwent successful emergent mechanical thrombectomy in the right middle cerebral artery with extended thrombolysis in cerebral infarction (eTICI) grade 3 recanalization. She was subsequently placed on systemic anticoagulation and a follow-up catheter angiogram done six weeks later showed spontaneous resolution of thrombus in the right vertebral artery with moderate residual stenosis (blue arrow).

**Figure 5:**
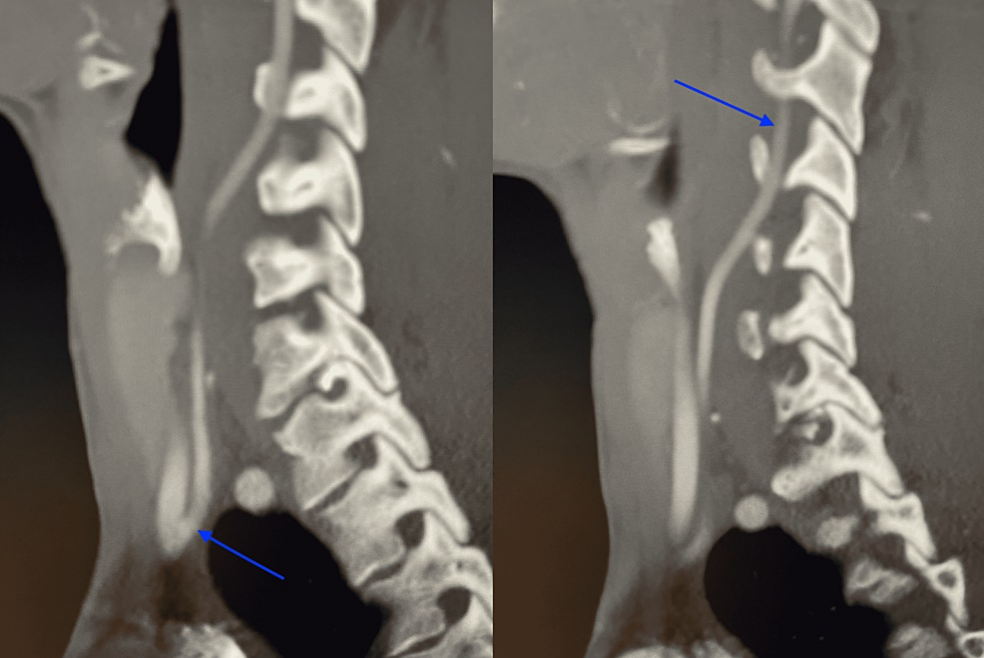
Follow-up CT angiogram of the head and neck showing restored patency of the right vertebral artery A follow-up CT angiogram of the head and neck performed after four months showed resolution of the dissection and restored patency of the right vertebral artery (left arrow). The right vertebral artery can be seen traversing through the transverse foramina of the cervical spine (right arrow).

The patient underwent successful emergent mechanical thrombectomy in the right MCA with extended thrombolysis in cerebral infarction (eTICI) grade 3 recanalization (Figure 4). She made a full recovery with no residual neurologic deficits. A follow-up MRI of the brain showed a small right basal ganglia infarct with no hemorrhagic conversion. The patient was subsequently placed on systemic anticoagulation and a catheter angiogram six weeks later showed spontaneous resolution of thrombus in the right vertebral artery with moderate residual stenosis (Figure 4). A follow-up CT angiogram of the head and neck was performed four months after the catheter angiogram, which showed resolution of the dissection and restored patency of the right vertebral artery (Figure 5). After extensive workup including a negative hypercoagulability panel, there was no other cause for the MCA stroke other than the dissection of the anomalous right vertebral artery. Although a clear cause-and-effect relationship cannot be confirmed, the dissection of the right vertebral artery is likely related to the patient’s right MCA stroke. The constellation of neck trauma, clinical symptoms, and CT angiography/angiogram/ultrasound findings led to the diagnosis of a right MCA stroke likely secondary to a dissection of an anomalous right vertebral artery.

## Discussion

In most cases, the paired vertebral arteries directly arise from the respective subclavian arteries and supply the brainstem and cerebellum [3]. Vertebral artery dissection is a result of blood entering the media through a tear in the intima of the vertebral artery and would lead to posterior circulation symptoms such as dizziness, visual disturbances, and gait problems [3].

However, our patient had an extremely rare anatomic variant with an anomalous right vertebral artery originating from the right proximal CCA. With neck manipulation from chiropractor therapy and vigorous exercise, she suffered a vertebral artery dissection and subsequent right MCA stroke leading to a left hemiparesis and speech deficits.

Variations in vertebral artery origin are all considerably rare but have been reported more commonly on the left side [4]. The most common anomaly overall is a four-vessel arch with a direct origin of the left vertebral artery off the aorta, which occurs in approximately 6% of the population [5]. Anomalous origins of the right vertebral artery can be divided into three types: aortic origin (0.05% incidence), common carotid/brachiocephalic origin (0.18-0.37%), and dual origin (0.04-0.4%) [6]. The common carotid/brachiocephalic origin is usually associated with an aberrant right subclavian artery arising directly from the aortic arch distal to the left subclavian artery and traversing posterior to the trachea and esophagus, which was seen in our patient [6]. When there is an associated aberrant right subclavian artery, the incidence of an anomalous right vertebral artery arising from the right CCA is much higher at 9.8% [1].

## Conclusions

The clinical entity of an anomalous right vertebral artery arising from the CCA with an aberrant right subclavian artery has been previously reported. It is usually an incidental finding on imaging; however, we present an interesting patient with right vertebral artery dissection in association with right MCA stroke. Although a clear cause-and-effect relationship cannot be confirmed, the dissection of the right vertebral artery is likely related to the patient’s right MCA stroke. Follow-up CT angiogram can be considered to monitor for disease progression or if symptoms reoccur.
